# Retraction Note: Defensive proclivity of bacoside A and bromelain against oxidative stress and AChE gene expression induced by dichlorvos in the brain of *Mus musculus*

**DOI:** 10.1038/s41598-026-47117-1

**Published:** 2026-04-07

**Authors:** Renu Bist, Bharti Chaudhary, D. K. Bhatt

**Affiliations:** 1 Department of Zoology, University of Rajasthan, Jaipur, Rajasthan 302004 India; 2 Department of Bioscience and Biotechnology, Banasthali University, Banasthali, Rajasthan 304022 India; 3 Department of Zoology, Mohanlal Sukhadia University, Udaipur, Rajasthan 313001 India

Retraction of: *Scientific Reports* 10.1038/s41598-021-83289-8, published online 11 February 2021

The Editors have retracted this Article.

After publication, concerns were raised regarding Figures 4A and 6A, specifically that the marker lanes of these Western blot images appear to overlap. The Editors requested all original raw data underlying the Article, but the Authors were not able to provide either the original raw images, the replicate images, nor the associated quantification data. The first author also stated that some of the data was lost. The Editors therefore no longer have confidence in the reliability of the data reported in the Article.

None of the Authors responded to correspondence from the Editors about this retraction notice.

